# The effect of intraoperative lidocaine infusion on opioid consumption and pain after totally extraperitoneal laparoscopic inguinal hernioplasty: a randomized controlled trial

**DOI:** 10.1186/s12871-020-01054-2

**Published:** 2020-06-03

**Authors:** Anup Ghimire, Asish Subedi, Balkrishna Bhattarai, Birendra Prasad Sah

**Affiliations:** 1 Department of Anesthesiology, Nepal Mediciti Hospital, Lalitpur, Nepal; 2 Department of Anesthesiology & Critical Care Medicine, BP Koirala Institute of Health Sciences, Dharan, Nepal

**Keywords:** Inguinal hernia, Laparoscopy, Lidocaine, Opioid analgesic, Postoperative pain

## Abstract

**Background:**

As a component of multimodal analgesia, the administration of systemic lidocaine is a well-known technique. We aimed to evaluate the efficacy of lidocaine infusion on postoperative pain-related outcomes in patients undergoing totally extraperitoneal (TEP) laparoscopies inguinal hernioplasty.

**Methods:**

In this randomized controlled double-blind study, we recruited 64 patients to receive either lidocaine 2% (intravenous bolus 1.5 mg. kg ^− 1^ followed by an infusion of 2 mg. kg^− 1^. h^− 1^), or an equal volume of normal saline. The infusion was initiated just before the induction of anesthesia and discontinued after tracheal extubation. The primary outcome of the study was postoperative morphine equivalent consumption up to 24 h after surgery. Secondary outcomes included postoperative pain scores, nausea/vomiting (PONV), sedation, quality of recovery (scores based on QoR-40 questionnaire), patient satisfaction, and the incidence of chronic pain.

**Results:**

The median (IQR) cumulative postoperative morphine equivalent consumption in the first 24 h was 0 (0–1) mg in the lidocaine group and 4 [1–8] mg in the saline group (*p* < 0.001). Postoperative pain intensity at rest and during movement at various time points in the first 24 h were significantly lower in the lidocaine group compared with the saline group (*p* < 0.05). Fewer patients reported PONV in the lidocaine group than in the saline group (*p* < 0.05). Median QoR scores at 24 h after surgery were significantly better in the lidocaine group (194 (194–196) than saline group 184 (183–186) (*p <* 0.001). Patients receiving lidocaine were more satisfied with postoperative analgesia than those receiving saline (*p* = 0.02). No difference was detected in terms of postoperative sedation and chronic pain after surgery.

**Conclusions:**

Intraoperative lidocaine infusion for laparoscopic TEP inguinal hernioplasty reduces opioid consumption, pain intensity, PONV and improves the quality of recovery and patient satisfaction.

**Trial registration:**

ClinicalTrials.gov- NCT02601651. Date of registration: November 10, 2015.

## Background

Inadequate pain relief after surgery causes undesirable effects. On the other hand, excessive use of opioids produces several adverse effects and might delay recovery [1, 2]. Therefore, a multimodal analgesia regimen is recommended in the perioperative setting as it provides superior analgesia and reduces opioid requirement [3]. Intravenous (IV) lidocaine is a widely studied drug for multimodal analgesia. IV lidocaine at the doses between 1.5–3 mg. kg^− 1^. h^− 1^ produces analgesic, anti-hyperalgesic, and anti-inflammatory effects [4]. Besides, a low dose of lidocaine is relatively safe and more feasible for perioperative use [4–7]. Additional benefits of lidocaine infusion include a reduction in the incidence of postoperative nausea and vomiting, early return of bowel motility and improved quality of recovery [8].

Several studies have shown that perioperative lidocaine infusion reduces postoperative pain intensity and opioid consumption, while others have found lidocaine to be ineffective [8]. These inconsistent findings may be due to variation in surgical procedure, dose and duration of lidocaine infused. Interestingly, a current update from Cochrane based meta-analysis found a weak evidence for IV lidocaine compared to placebo on early postoperative pain scores and overall opioid requirements [9]. On the contrary, other recently published meta-analyses have shown improvement in postoperative pain-related outcomes with lidocaine infusion during laparoscopic clolecystectomy [10, 11].

Although lidocaine infusion was effective for postoperative analgesia in open inguinal hernia surgery [12], its use has not been reported in totally extraperitoneal (TEP) laparoscopic inguinal hernioplasty. Therefore, the primary objective of our study was to compare the effects of intraoperative lidocaine infusion on postoperative opioid consumption following TEP laparoscopic inguinal hernioplasty.

## Methods

This prospective randomized double-blind clinical trial was conducted at the BP Koirala Institute of Health Sciences (BPKIHS) from December 2015 to March 2017. Ethical approval for this study (Ref No. IRC/520/015) was provided by the Institutional review committee of BPKIHS, Dharan, Nepal (Member secretary Dr. Ashish Shrestha) on 24 June 2015. Before enrollment of patients, the trial was registered by the principal investigator (AG) at clinicaltrials.gov (Ref No. NCT02601651). The trial was conducted according to Good Clinical Practice and the Consolidated Standards of Reporting Trials (CONSORT) guidelines.

Patients were screened for eligibility (AG) during the pre-anesthetic visit at the in-patient-unit, the night before surgery. Male patients aged between 18 and 65 years, of ASA physical status I–II, planned for laparoscopic TEP repair of the inguinal hernia were eligible. Patients were excluded if they were obese, unable to comprehend the pain assessment scale, allergic to local anesthetics, on pain medication or anti-arrhythmic drugs, or had, psychiatric disorders, cardiac arrhythmia, hepatorenal disease or epilepsy.

After obtaining written informed consent, all eligible participants were randomly assigned, in a 1:1 ratio, to receive either lidocaine (intervention) or normal saline (placebo comparator) infusion. The anesthesia supporting staff created the trial-group assignment from the computer-based randomization list, which remained secured in sequentially numbered sealed opaque envelopes and concealed until after enrollment.

On the day of surgery, an anesthesia assistant not involved in the study prepared the drug solution after breaking the codes. Patients received one of the two assigned study medications just before the induction of anesthesia: Lidocaine group received an IV bolus of 1.5 mg. kg^− 1^ lidocaine (Lox 2%®, Neon pharmaceuticals limited, Mumbai, India) followed by a continuous infusion of 2 mg. kg^− 1^. h^− 1^ until the tracheal extubation; The saline group received an equal volume of IV 0.9% normal saline (NS) bolus followed by a continuous infusion. Patients, attending anesthesiologists, and the investigator who collected the data and assessed the outcomes were unaware of the trial-group assignment.

Patients received no premedication. During the pre-anesthetic visit, they were educated on the numeric pain rating scale (NRS, 0–10 cm) for postoperative pain, where 0 is no pain and 10 is the worst imaginable excruciating pain. In the operating room, standard monitoring was applied. Just before the induction of anesthesia, patients received the study drug, according to the group allocation. Anesthesia was induced with IV fentanyl 1.5 μg. kg^− 1^ and propofol 2–2.5 mg. kg^− 1^ till the cessation of verbal response and the tracheal intubation was facilitated with vecuronium 0.1 mg. kg^− 1^ IV. The lungs were mechanically ventilated in volume control mode, maintaining the end-tidal carbon dioxide (ETCO_2_) between 35 and 45 mmHg.

Intravenous paracetamol 1 g was administered for 15 min after tracheal intubation. Pre-incisional infiltration in the three trocar sites was done with 2 ml of 0.25% bupivacaine. Anesthesia was maintained with an air / oxygen mixture (inspired oxygen fraction 0.40) and isoflurane, adjusting the end-tidal concentration of isoflurane to maintain mean arterial pressure (MAP) within 20% of the baseline. IV fentanyl 0.5 μg. kg^− 1^ was supplemented intraoperatively if MAP and heart rate increased by 20% from the baseline after ensuring adequate end-tidal concentration of isoflurane, neuromuscular blockade and targeted range of ETCO_2_. The adequate neuromuscular blockade was achieved with supplemental doses of vecuronium IV bolus after observing curare notch in capnograph. Any episode of intraoperative hypotension (MAP < 65 mmHg) and bradycardia (heart rate < 50 beats. min^− 1^) was treated with ephedrine 5 mg and atropine 0.4 mg IV respectively.

An experienced surgeon performed the TEP laparoscopic surgery for inguinal hernia repair as described elsewhere [13]. Ketorolac 30 mg IV was administered at the end of surgery and scheduled to be given at 8 h intervals. The residual neuromuscular block was reversed with IV neostigmine 0.05 mg. kg^− 1^ and glycopyrrolate 0.01 mg. kg^− 1^. Following successful tracheal extubation, the study drug was discontinued and the patient was transferred to the postanesthesia care unit (PACU).

The blinded investigator assessed the postoperative outcomes. The primary outcome was total IV morphine equivalent consumed in the first 24 h. Secondary outcomes were postoperative pain scores (NRS) at rest and on movement, sedation scores recorded using a 5-point scale (0 = alert, 1 = arouses to voice, 2 = arouses with gentle tactile stimulation, 3 = arouses with vigorous tactile stimulation, 4 = lack of responsiveness) [14], the incidence of PONV using a 3-point scale (0 = none, 1 = nausea, 2 = vomiting), time to the first perception of pain (min), time to first void (h), adverse events (lightheadedness, tinnitus, perioral numbness, arrhythmia), quality of recovery based on QoR-40 questionnaire [15] at 24 h after surgery, patient satisfaction for postoperative pain relief using a five-point Likert scale at 24 h following surgery (1-highly satisfied, 2-satisfied, 3-neutral, 4-not satisfied, 5-strongly dissatisfied) and the incidence of chronic post-surgical pain (CPSP) at 3 months.

Pain and sedation scores were assessed at PACU (on arrival, 15 min, 30 min, 1 h, 2 h) and surgical unit (4 h, 6 h, 8 h, 12 h, 24 h). If the NRS score for pain was > 3 at rest, morphine 1 mg IV bolus was administered in the PACU, and repeated at 5 min interval until NRS was ≤3. After 2 h of the stay in the PACU, the patients were transferred to the ward. In the surgical unit, tramadol 50 mg IV was administered for NRS score > 3 and 50 mg was repeated at 10 min interval, up to a maximum dose of 300 mg in the first 24 h for maintaining VAS score for pain ≤3. The amount of tramadol consumed was converted to an equivalent dose of morphine from an online dose equivalent calculator (www.clincalc.com/Opioids). Ondansetron 4 mg IV was administered for persistent nausea (lasting > 5 min) or vomiting. CPSP was defined as pain that developed after a surgical procedure and persisted at least 3 months after surgery [16]. For this, the blinded investigator contacted the patients via telephone at 3 months after surgery. They were asked to answer the following question: Do you feel any pain in the operated area?

The sample size calculation was based on the study by H Kang on postoperative opioid consumption between the lidocaine infusion group and the placebo group in open inguinal hernia surgery [12]. Using an online statistical calculator (G power® version 3.0.1), an estimated sample size of 29 patients in each study group achieved a power of 80% to detect a Cohen’s d effect size of 0.76 in the primary outcome measure of opioid consumption, assuming a type I error of 0.05. With an anticipated 10% drop-out, a total of 64 patients were enrolled.

The data were entered into excel software and analyzed using STATA version 13.0 (Stata Corporation, College Station, TX, USA). Histograms and the Shapiro-Wilk test was used to check the normality of the data. Normally distributed data were compared using a 2-tailed t-test for independent samples. Non-normally distributed data were analyzed using the Mann-Whitney U test. For ordinal data, the Kruskal-Wallis test was applied. Chi-square test or Fischer’s exact test was used for analyzing the categorical variables as appropriate. The finding with an associated *p*-value less than 0.05 was considered as statistically significant.

## Results

**Fig. 1:**
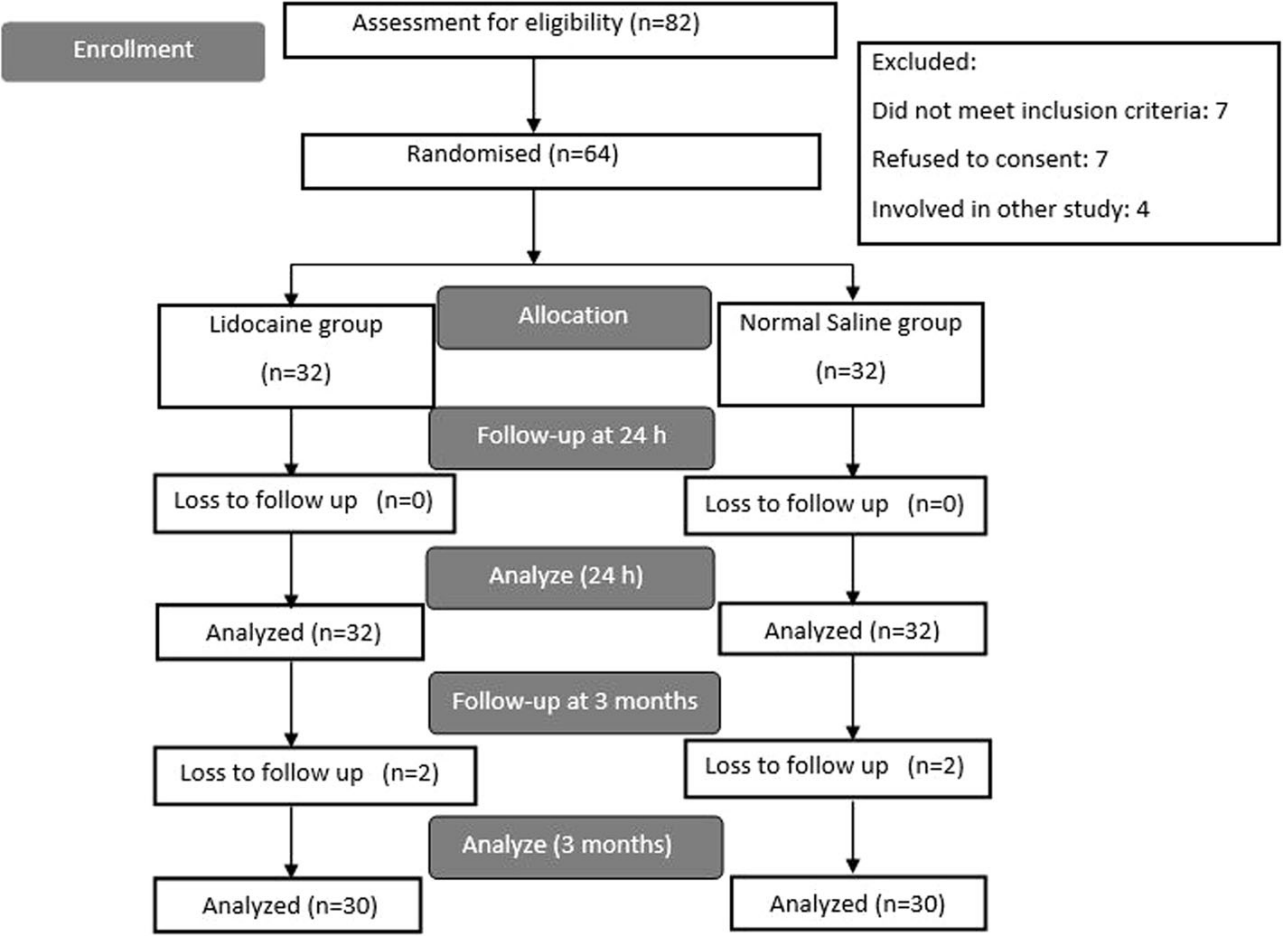
CONSORT diagram of patient recruitment

Of the 82 screened patients, 18 patients were excluded (Fig. 1). Two patients in each group could not be traced during follow-up in 3 months. All outcomes were analyzed with the intention-to-treat principle. The demographics and surgical characteristics between the two groups did not reveal any significant differences (Table 1). The median (IQR) intraoperative fentanyl consumption was significantly less in the lidocaine group 0(0–0) μg vs. 20 (0–30) μg in the saline group (*p* < 0.001).

**Table 1:** Patient characteristics and surgical profiles of patients

Variables	Lidocaine group (*n* = 32)	Normal saline group (*n* = 32)	*P-*value
Age (years)	40 (30–52)	43 (33–52)	0.61
ASA PS (1/2)	28/4	27/5	0.71
BMI (kg/m^2^)	23.02 ± 2.85	22.01 ± 2.02	0.10
Surgical site: Unilateral/Bilateral	25/7	23/9	0.56
Mesh fixation (Yes/No)	31/1	32/0	0.50
Duration of surgery (min)	60 (48–90)	75 (60–90)	0.49
Intraoperative fentanyl supplement (μg)	0 (0–0)	20 (0–30)	< 0.001

Notes: Values are median (IQR), mean (SD), number.

Abbreviations: *BMI* body mass index; *ASA PS* American society of Anesthesiologist physical status

**Fig. 2:**
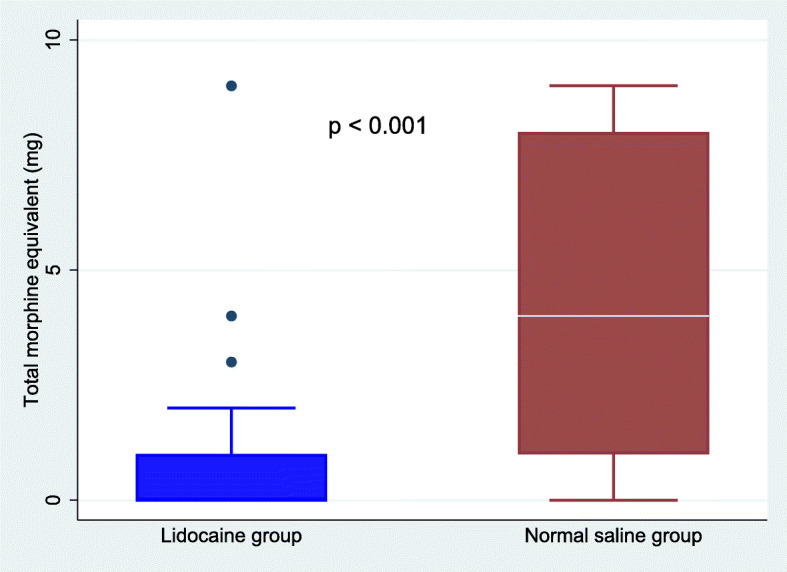
Total morphine equivalent for 24 h postoperatively in patients receiving lidocaine and saline. Data are presented as median and interquartile range

**Fig. 3:**
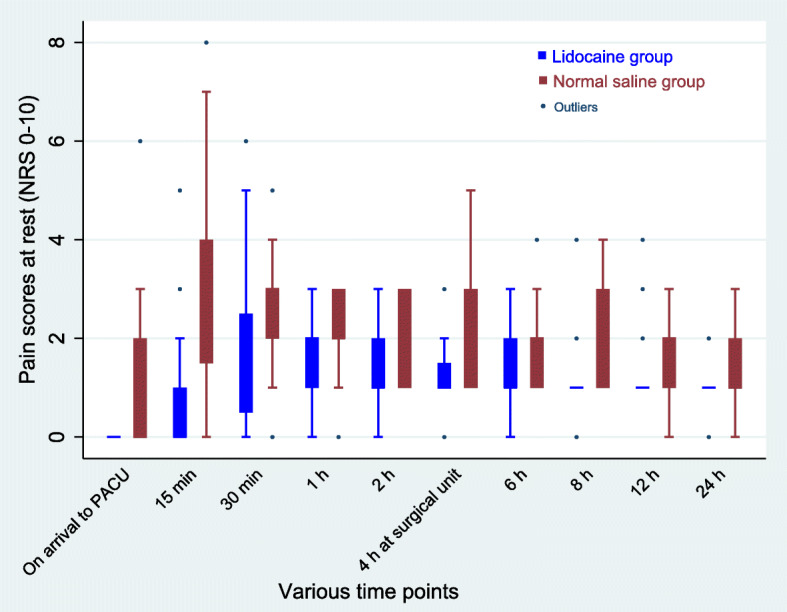
Postoperative numerical rating pain (NRS) scores at various time points at rest. Data are median with error bars showing interquartile range. Significant difference between the groups was detected at all-time points (*p* < 0.05)

**Fig. 4:**
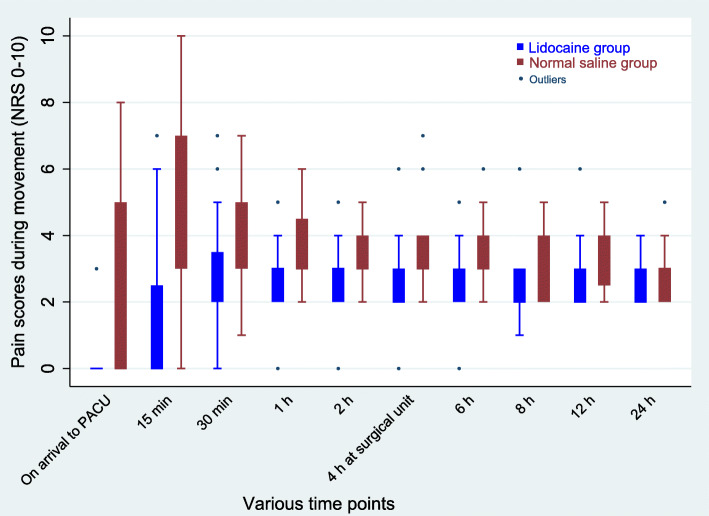
Post-operative numerical rating pain scores (NRS) at various time points during movement. Data are median with error bars showing interquartile range. Significant difference between the groups was detected at all-time points (*p* < 0.05)

The cumulative median IV morphine equivalent consumption at 24 h postoperatively was significantly reduced in the lidocaine group than in the saline group (Fig. 2). The median morphine requirement in PACU was 0 (0–1) mg in the lidocaine group compared with 2 (0–4) mg in the saline group (*p* = 0.003). In the surgical unit, patients consumed a lesser median (IQR) tramadol in the lidocaine group, 0 (0–0) mg compared with the saline group 0 (0–50) mg (*p* < 0.001). The median NRS scores at rest and during movement were significantly lower in the lidocaine group than in the saline group at all time points after surgery (Figs. 3 & 4). The time to the first perception of pain was longer in those receiving lidocaine (median 30 min (15–30) compared with those receiving NS (median 10 min (0–15); *p* < 0.001).

A significant number of patients in the saline group had PONV and needed antiemetic compared to the lidocaine group (Table 2). Postoperative sedation scores were comparable between the two groups. Postoperative quality of recovery and patient satisfaction with postoperative pain relief was better in those receiving lidocaine (Table 2). No sign/symptoms related to lidocaine toxicity were observed. One patient in the lidocaine group developed intraoperative hypotension and bradycardia which was managed with ephedrine 5 mg and atropine 0.4 mg intravenously. When assessed in 3 months after surgery, two (7%) patients in the lidocaine group developed CPSP compared to four (13%) in the placebo group (*p* = 0.67).

**Table 2:** Postoperative outcomes

	Lidocaine group (*n* = 32)	Saline group (*n* = 32)	*p*-value
Nausea	5 (16%)	14 (44%)	0.01
Vomiting	2 (6%)	8 (25%)	0.04
Antiemetic needed	3 (9%)	11 (34%)	0.01
Time to first void; h	3 (2–4)	3 (3–4)	0.18
Quality of recovery;QoR-40 scores	194 (194–196)	184 (183–186)	< 0.001
Patients with satisfaction scores 1/2/3/4/5^a^	6/17/9/0/0	2/13/17/0/0	0.02

Notes: Values are number (proportion), or median (IQR)

^a^Satisfaction scores for postoperative pain relief, 1-Highly satisfied, 2-Satisfied, 3-Neutral, 4-Not satisfied, 5-Strongly dissatisfied

## Discussion

Our study showed that intraoperative infusion of low dose lidocaine decreased postoperative opioid requirement and pain intensity in comparison with normal saline in patients undergoing laparoscopic TEP inguinal hernia surgery. Patients receiving lidocaine had fewer occurrences of PONV, a better quality of recovery and were more satisfied with postoperative pain relief than those receiving saline. Patients complained of pain later in the lidocaine group than the saline group. No significant difference was observed for postoperative sedation and the incidence of chronic pain in 3 months.

It is well-established that lidocaine acts on voltage-gated sodium channels when administered locally for peripheral nerve block. However, at lower concentration systemic lidocaine is insufficient to produce direct analgesia solely by blocking the neuronal sodium channels [17]. Although it is not fully understood how intravenous lidocaine produces analgesia, several potential mechanisms have been elucidated. Intravenous lidocaine increases acetylcholine concentration at the spinal level through an activation of both muscarinic and nicotinic receptors, and thereby prolongs the pain threshold [18]. Also, by activating central glycine (an inhibitory neurotransmitter) receptor, systemic lidocaine inhibits glutamate-induced excitatory response on the wide dynamic response in the spinal neurons [19]. The anti-hyperalgesic effect of IV lidocaine is due to blockade of NMDA receptor signaling and it is mediated indirectly by inhibition of the protein kinase C pathway [20]. In addition to this, systemic lidocaine has anti-inflammatory properties as a decline in pro-inflammatory cytokines is observed in patients receiving lidocaine infusion [21–23]. Because perioperative pain is linked to an inflammatory process, modulation of this phenomenon with the administration of systemic lidocaine could significantly reduce pain. Another relevant question is to explain how the intraoperative administration of IV lidocaine does reduces opioid and pain scores beyond its infusion period. This could be due to its action on various receptors and signal cascades that produces an anti-nociceptive, anti-hyperalgesia and anti-inflammatory effects [8].

Because of its influence in several pain pathways, systemic lidocaine is widely investigated adjuvant in the regimen of multimodal analgesia to reduce postoperative opioid consumption and pain. Although the majority of studies have demonstrated the analgesic effect of lidocaine, several other trials failed to confirm it. A recently updated Cochrane review in 2018 has provided a much-needed insight on the analgesic property of systemic lidocaine [9]. Random-effects meta-analysis from the same review on overall total postoperative opioid consumption favored lidocaine compared to the placebo (standardized mean difference (SMD) − 4.52 (mg, morphine equivalents (MEQ), 95%CI − 6.25 to− 2.79, *p* < 0.001; I^2^ = 73%; 40 studies, 2201 participants). The results of our study also indicated a similar reduction in total postoperative opioid consumption in the first 24 h after surgery in the lidocaine group compared to the saline group (median difference of − 4 mg morphine equivalents), despite using multimodal analgesia in both the groups.

Further, the aforementioned meta-analysis [9] demonstrated reduced pain scores at rest (“early time points”- in the PACU or 1 to 4 h postoperatively) in the lidocaine group compared to the control group (SMD − 0.50, 95% CI − 0.72 to− 0.28; Test for overall effect: Z = 4.41 (*P* < 0.0001). This was equivalent to an average pain reduction between 0.37 cm and 2.48 cm on a VAS 0 to 10 cm scale in the lidocaine group. Likewise, at intermediate time points (24 h postoperatively) the standardized mean pain score at rest in the lidocaine group was 0.14 lower (95% CI − 0.25 to − 0.04; Test for overall effect: Z = 2.63 (*P* = 0.0086). This was equivalent to an average pain reduction in the lidocaine group between 0.48 cm and 0.10 cm on a VAS 0 to 10 cm scale. These results showed that lidocaine exerted a clinical difference of at least 1 cm on a 0–10 VAS scores for pain at rest during early time points (1 to 4 h); however, this difference was not observed at intermediate (24 h) time points. We too observed statistically significant difference in pain scores up to 24 h postoperatively, while the clinical difference of approximately 1 cm in NRS scores at rest was observed only up to 1 h.

Due to substantial heterogeneity between studies, the authors of the same meta-analysis performed a sub-group analysis based on type of surgery, duration and dose of lidocaine infusions [9]. In the older version (Cochrane review, 2015) there was a clear beneficial effect in terms of pain reduction in laparoscopic abdominal surgery compared to open abdominal surgery [6]. However, in the current updated version, no significant difference was observed, although the trend was towards a beneficial effect for abdominal laparoscopic surgery [9].

The optimal dose and time to terminate lidocaine infusion are still an unsolved issue. We had limited the duration of lidocaine infusion until the patients trachea was extubated due to a lack of dedicated infusion pumps and monitoring at the surgical unit. One might hypothesize that longer infusions would lead to more lasting analgesia but studies are yet to confirm this. The current meta-analysis (2018) had categorized the studies according to the usage of low (< 2 mg.kg^− 1^. h^− 1^) and high (≥ 2 mg.kg^− 1^ h^− 1^) lidocaine doses in combination with either short (until the end of surgery or until PACU) or long (≥ 24 h postoperatively) duration of infusion [9]. However, they did not find any difference in outcomes when the dose or duration of the infusion was compared. A well designed randomized comparative study with a large sample size is needed to explore whether the continuation of systemic lidocaine infusion beyond the surgical period is effective.

In our study, fewer patients receiving lidocaine complained PONV compared to those receiving saline infusions. Similar to our finding, the Cochrane meta-analysis (2018) reported a significantly lower frequency of nausea in the lidocaine group than in the control group, but the vomiting rates did not differ [9]. Although, there is an association between lidocaine therapy and reduction in PONV, it may not reflect a causal relationship. The most likely explanation for this association is related to lidocaine’s opioid-sparing effects.

Recently, there is a growing interest in patient-reported outcomes such as postoperative QoR and patient satisfaction. We observed better recovery profiles at 24 h of surgery in the lidocaine group as evident from the QoR scores. Similar to our study, De Oliveira and his colleagues reported greater QoR-40 scores at 24 h with perioperative lidocaine infusion for laparoscopic abdominal surgery [24, 25]. Likewise, in our study patient satisfaction was better in lidocaine than saline group and no patient expressed dissatisfaction over the intervention. The current meta-analysis also supports this finding by revealing higher satisfaction scores in patients receiving lidocaine compared to placebo group (SMD 0.76, 95% CI 0.46 to 1.06; I^2^ = 0%; 6 studies, 306 participants) [9]. Further, perioperative lidocaine infusion reduces the length of hospital stay as compared to the placebo. We considered this outcome as a limitation in our study because all our participants were required to stay in the hospital for 24 h after surgery. In terms of patient-reported outcomes, it would be interesting to explore the influence of perioperative lidocaine on the enhancement of recovery profiles, especially after major abdominal surgeries in future trials. A more recent meta-analysis focused on CPSP (total 6 trials included: 4 mastectomies, 1 thyroidectomy, 1 nephrectomy) found that systemic lidocaine administration reduces the development of CPSP [26]. As our study was not powered enough to detect the protective effect of lidocaine on CPSP after laparoscopic TEP, we would not like to draw any conclusion. This could be explored in a larger, multi-centric trial with CPSP as a primary outcome.

## Conclusions

In summary, intraoperative lidocaine infusion decreases overall opioid requirement and postoperative pain intensity in patients undergoing laparoscopic TEP inguinal hernioplasty. It also lowers the incidence of PONV, improves the quality of recovery and patients satisfaction without any sedative effect.

## Data Availability

The datasets used and/or analysed during the current study are available from the corresponding author on reasonable request.

## Abbreviations

TEP: Totally extraperitoneal
PONV: Postoperative nausea and vomiting
QoR: Quality of recovery
IQR: Interquartile range
IV: Intravenous
BPKIHS: BP Koirala Institute of Health Sciences
ASA: American Society of Anesthesiologists
NS: Normal Saline
NRS: Numerical rating scale
ETC0_2_: End-tidal carbondioxide concentration
MAP: Mean arterial pressure
PACU: Post anesthesia care unit
CPSP: Chronic post-surgical pain
SMD: Standardized mean difference
MEQ: Morphine equivalent
VAS: Visual analogue scale
